# Curriculum Innovations: Enhancing Medical Student Neuroscience Training With a Team-Based Learning Curriculum

**DOI:** 10.1212/NE9.0000000000200037

**Published:** 2023-01-05

**Authors:** Christopher G. Tarolli, Ralph F. Józefowicz

**Affiliations:** From the Department of Neurology (C.G.T., R.F.J.), and Center for Health + Technology (C.G.T.), University of Rochester, NY.

## Abstract

**Background and Problem Statement:**

Neurophobia, the fear of, discomfort with, and dislike of clinical neurology, is frequently due to poor experiences in preclinical neuroscience education among medical providers. We developed, implemented, and assessed a curricular innovation using clinician-educators and team-based learning (TBL) with the goals to demonstrate clinical relevance in neuropathology, enhance student engagement in neuropathology education, and promote direct application of knowledge.

**Methods and Curriculum Description:**

We identified an underperforming neuropathology curriculum within the second-year medical student neuroscience course at the University of Rochester School of Medicine and Dentistry and implemented a traditional TBL curriculum to deliver this content. In addition, we transitioned to primarily clinician-led lectures in the neuropathology curriculum. We assessed student opinions of the curricular changes though end-of-course feedback, the implementation of a novel survey, and semistructured interviews with students. We assessed outcomes on the course final examination and overall course performance, comparing student performance in the preimplementation phase (year 2020–2021) with that in the postimplementation phase (year 2021–2022) using a 2-sample *t* test.

**Results and Assessment:**

Student opinions of the curricular changes were positive on the end-of-course evaluation (79.4% rated TBL as good or excellent) and novel survey (89%–96% of students rated the portions of the curriculum positively). Themes identified in free text responses and through qualitative interviews included an appreciation of the streamlined course content and a sense that the various sessions within the neuropathology curriculum effectively reinforced learning. Student performance on the final examination was similar in the preimplementation vs postimplementation phases (81.2% correct vs 80.3% correct; *p* = 0.37). Performance on the neuropathology subsection of the final examination was also similar among the 2 cohorts (82.6% correct vs 83.9% correct; *p* = 0.36).

**Discussion and Lessons Learned:**

We demonstrate the feasibility and utility of a transition to primarily neurologist and neurosurgeon-led lectures and the implementation of a TBL curriculum within a neuroscience course. While we report data from implementation at a single center, these results have potential relevance to other courses, given our demonstration that TBL is a useful method to deliver neuroscience learning, nonpathologist lecturers can effectively provide neuropathology education, and a small number of educational faculty can be engaged to deliver this material.

## Introduction and Problem Statement

### Problem Statement

Nonengaging neuroscience teaching with a failure to demonstrate relevance to clinical neurology can result in the development of neurophobia among medical students.^1^ In this study, we propose a curricular innovation using clinician-educators and team-based learning (TBL) to enhance poorly rated neuropathology education in a medical school neuroscience course.

The global burden of neurologic disease is growing, and providers across medical specialties will interact with and manage patients with neurologic conditions.^2,3^ Given this, it is essential that medical trainees develop competence and confidence in the care of patients with neurologic disease. Unfortunately, medical education today falls short on this imperative. Among US medical graduates, neurology clerkship quality is rated the second lowest across core rotations, and only approximately 2.5% of graduates choose to enter neurology, a number that has remained static over the past 10 years.^4^

**Figure 1:**
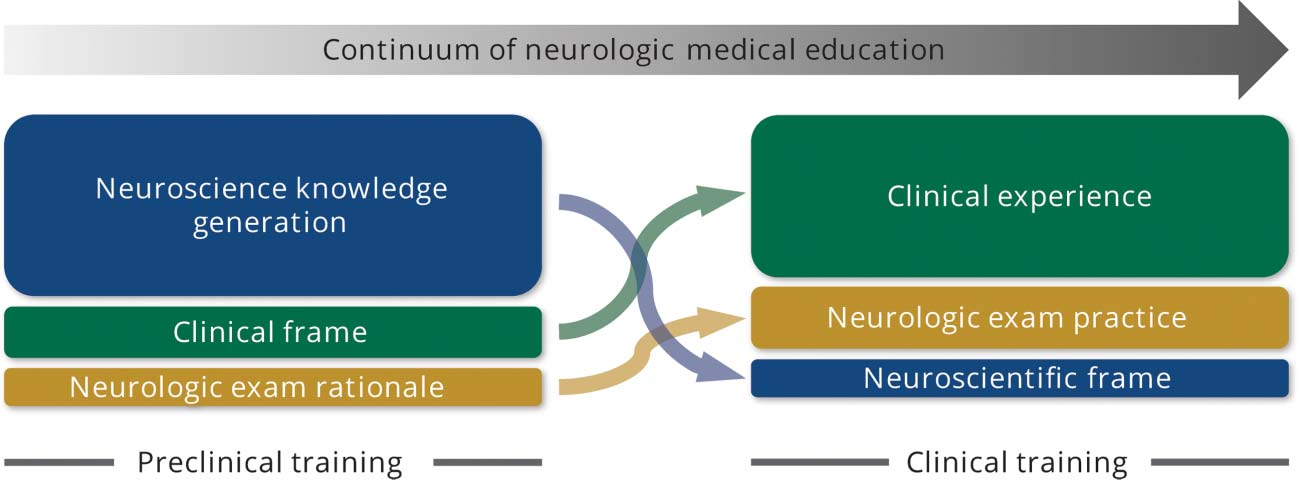
Framework for the Implementation of a Continuum of Neurologic Education in Undergraduate Medical Education That Focuses on Neuroscience Knowledge Generation Within a Clinical Frame in the Preclinical Years and the Development of Clinical Expertise With a Grounding in Neuroscientific Principles in Clinical Training

Neurophobia is a principal driver of these outcomes and is often caused by poor experiences in the preclinical years with students unable to apply knowledge of basic sciences to their clinical experiences.^1^ There is an increasing literature on methods to mitigate neurophobia in preclinical training with clear benefit from shifting to active learning methods; engaging clinicians in neuroscience training; and demonstrating the application of neuroscientific principles to clinical neurology.^5-8^ With these goals in mind, neurologic educators should strive to establish a continuum of neurologic education with neuroscience learning grounded in clinical neurology and clinical training grounded in neuroscientific principles (Figure 1).

TBL is an established didactic method in medical education that uses a constructivist paradigm to facilitate knowledge acquisition and application.^9,10^ Learners complete self-directed learning at home, participate in in-class readiness tests, and complete team-based case review in a large group setting to apply the recently acquired knowledge.^9,11^ Group learning is overseen by a faculty facilitator, and a single facilitator can lead a group of 100 students or more. Given these features, TBL is an optimal method to facilitate neuroscience learning. The scaffolded approach facilitates low-level knowledge acquisition (remembering and understanding) with the asynchronous material and allows students to use higher-level knowledge attainment skills (applying, analyzing, and evaluating) in active sessions led by a clinician facilitator, where they use the knowledge gained in the prework.^12,13^ This facilitates greater retention of knowledge, provides frequent and timely feedback to students, and allows opportunities for informal peer evaluation in the group readiness test and in case reviews.^14^ There is some literature on the use of TBL in neuroscience education, though the use of a formal TBL curriculum has primarily been described in premedical or international medical education.^15-21^

## Objectives

The preclinical neuroscience course at the University of Rochester School of Medicine and Dentistry uses many of the methods described earlier to mitigate neurophobia with outstanding outcomes in student feedback and performance on standardized examinations. However, the neuropathology curriculum was an outlier within the course largely due to a reliance on passive learning and redundancy across didactic experiences with lower opinions of neuropathology content compared with other components of the course. Course objectives in this portion of the curriculum focus on teaching students about the pathologic basis for nervous system disease and providing a sufficient background to permit knowledgeable participation in the care of individuals with neurologic disease. In this study, we describe the development and implementation of a novel neuropathology curriculum with the goals to achieve the following:

1. Demonstrate the clinical relevance of neuropathology with a transition to primarily neurologist and neurosurgeon-led lectures in the neuropathology curriculum.
2. Enhance student engagement in neuropathology education through the implementation of a TBL curriculum in neuropathology.
3. Promote direct application of knowledge through the implementation of a TBL curriculum in neuropathology.

## Methods and Curriculum

### Course Background

The preclinical neuroscience course at the University of Rochester School of Medicine and Dentistry is a 9-week course at the start of the second phase (year) of the 4-year medical school curriculum. The neuropathology curriculum extends over the final 4 weeks of the course, covering 12 topics in neuropathology with 1 hour of lecture and laboratory time each, totaling 24 hours of course time. Before implementation of the novel curriculum, a typical day of neuropathology learning consisted of 2 hours of lecture covering 2 topics, followed by a medium group (approximately 25 students) neuropathology laboratory time (Table 1). A neuropathologist provided 9 of the 12 hours of neuropathology lectures prior to implementation of the new curriculum, with the remaining sessions covered by the course director (2 hours) and an infectious disease specialist (1 hour). Laboratory sessions were run by a neuropathologist or one of the course directors (neurologists) with review of gross and histopathologic slides, as well as neuroimaging on relevant topics via slide presentations. There was substantial redundancy in the material presented in the lecture and laboratory portions of the teaching with the same disease processes, key learning points, and, at times, the same slides used between the 2 sessions. In addition, there was minimal active learning in either the lectures or the laboratory sessions.

**Table 1:** Structure of Neuropathology Learning Days Preimplementation (1A) and Postimplementation (1B) of the Curricular Changes

1A: Preimplementation structure			1B: Postimplementation structure		
Time	Description/schedule		Time	Description	
8:00–9:00	Lecture	Neuropathology topic 1 Lecturer: Neuropathologist	8:00–9:00	Lecture	Neuropathology topic 1 Lecturer: Neurologist/Neurosurgeon
9:00–10:00		Neuropathology topic 2 Lecturer: Neuropathologist	9:00–10:00		Neuropathology topic 2 Lecturer: Neurologist/neurosurgeon
10:00–11:00	Lab	Neuropathology laboratory topic 1 Format: Slide presentation	10:00–10:10	TBL (2 topics)	IRAT (8 questions)
			10:10–10:25		GRAT (8 questions)
11:00–12:00		Neuropathology laboratory topic 2 Format: Slide presentation	10:25–10:45		IRAT/GRAT review
			10:45–11:30		Application exercises
			11:30–11:50		Application exercise review

Abbreviations: GRAT = group readiness assessment test; IRAT = individual readiness assessment test; TBL = team-based learning.

### Curricular Changes

To reduce redundancy between the lecture and laboratory curricula, we invited neurologists and neurosurgeons from clinical specialties aligned with each neuropathology topic (e.g., a neuroimmunologist was invited to lecture on disorders of myelin) to provide the lectures. The directive for new lecturers was to update the lecture syllabi and to provide an overview of gross and histopathology on the topic with a strong clinical anchor and correlation. Invited faculty were selected based on course leadership knowledge of the faculty member's teaching abilities. A pathologist continued to provide an overview of neuropathology as the first lecture in the series, and 4 new faculty were added to the teaching roster with the remaining lectures given by faculty members who were already involved in other portions of the course. All invited faculty members accepted the invitation.

We converted the laboratory activities to a TBL format. Each TBL session lasted approximately 2 hours and covered 2 topics in neuropathology. TBL is used in other courses in the preclinical curriculum at the University of Rochester, and the session schedule is based on the format used in a formal TBL curriculum^9,22^; our session schedule was modeled after this, and a typical breakdown of the time in each session is summarized in Table 1. We elected to split the class of 104 students into 2 groups, with one group led by the course director (C.G.T.) and the other group led by the former course director (R.F.J.). Adult and child neurology chief residents who participate as small group leaders in the course assisted with the facilitation of the sessions.

Course faculty created syllabi for each TBL topic to serve as the presession material that students reviewed prior to TBL. Among the 12 topics, the course director (C.G.T.) created 8 new syllabi, repurposing content from the prior neuropathology laboratories and other prior course materials; lecturing faculty members provided new syllabi for 3 additional topics; the final prereading was created using excepts from the textbook, Escourolle and Poirier's *Manual of Basic Neuropathology*,^23^ which was available for free to students through the medical center library. A sample of one of the presession syllabi is included in eMethods 1 (links.lww.com/NE9/A10). The time required to review each syllabus was estimated to be approximately 30 minutes per topic.

Each TBL session starts with a 10-minute individual 8-question quiz (4 questions per topic) termed the individual readiness assessment test (IRAT), which is based on the presession materials (eMethods 1, links.lww.com/NE9/A10). The course director (C.G.T.) created the quiz questions, and the prior course director (R.F.J.) and neurology chief residents reviewed the questions for content. Questions were generally 5-option multiple choice questions, with each question framed in a clinical vignette; questions included a combination of gross and histopathologic images, as well as neuroimaging. Quiz implementation was completed and scored on the Blackboard Learn platform (Reston, VA), which houses all course materials. After the IRAT, students complete the same 8-question quiz termed the group readiness assessment test (GRAT) with their team of 5–6 students that remains static over the course; group responses are submitted by 1 member of each group via Blackboard. After the GRAT, the group facilitator reviews the 8 questions with each team lifting a placard corresponding with their answer. The time spent on review of each question is modified based on the unanimity of responses, with more time spent reviewing items where questions arise or disagreement is apparent.

Students spend the next 45 minutes completing 4 application exercises (2 per topic). Application exercises are 3- to 4-question extended clinical vignettes with intermixed multiple choice questions; application exercises also included a combination of gross and histopathologic images, as well as neuroimaging (eMethods 1, links.lww.com/NE9/A10). The course director created all application exercises, and the former course director, lecture faculty, and neurology chief residents reviewed the application exercises for content. After completing the application exercises, the group facilitator reviews each of the application exercise questions with each team lifting a placard corresponding to their response with a similar format to the IRAT/GRAT review.

### Outcomes and Analysis

Outcomes were assessed using Kirkpatrick model of training evaluation with a focus on reaction, learning, and results.^24^ Lecture quality was assessed based on end-of-course feedback. A random selection of 25% of the class was invited to review each lecture, with rating from 1 (poor) to 5 (excellent); students can additionally provide narrative comments on lecturers. We compared the mean rating for lecturing faculty in the implementation year to the mean lecture quality rating for the neuropathology lecture faculty in the prior academic year using a 2-sample *t* test. Because 2 neuropathology faculty lecturers provide a number of other lectures in the course, we conducted the analysis with and without their inclusion.

We assessed student opinions of TBL in multiple ways. First, we evaluated both quantitative and qualitative end-of-course feedback evaluations; session quality is rated on a 1 (needs much improvement) to 5 (excellent) scale, and students have the option to provide narrative feedback as well. In addition, we created a novel survey to assess student opinions of each component of the TBL session (prereading, IRAT/GRAT, application exercises, and review portions), facilitator quality, and overall impression via narrative comment. The survey was reviewed by faculty experts for content and pretested by 2 third year medical students with minimal changes made to improve question clarity.

The survey was administered via Research Electronic Data Capture, a secure web application that provides a toolset for effective data collection and management.^25^ Students were invited to participate via email. On clicking the survey link, students were presented with an information sheet prior to survey completion. Survey completion was anonymous. However, at the end of the survey, students had the option to agree to participate in an interview to discuss their opinion of the TBL sessions. If the student agreed to participate, they were redirected to another form where they could provide their contact information. This was not linked to their survey responses. There was no incentive offered for completing the survey or participating in an interview.

Interviews were scheduled and conducted by the course director, who followed a semistructured format with prewritten questions (eMethods 2, links.lww.com/NE9/A11); all interviews were recorded and lasted 30–45 minutes. We aimed to conduct approximately 10 interviews (∼10% class participation). All narrative feedback and qualitative interview responses were reviewed for common themes. We did not follow a coding or formal qualitative review process because the goal of the review was for session improvement, rather than a sociological assessment of student opinion.

We assessed the impact of the updates to the neuropathology curriculum on student outcomes in the course. Course assessments include 3 in-house multiple choice examinations (total 300 questions), and a 140-question National Board of Medical Examiners (NBME) subject test in neuroscience with questions selected by the course director. We compared final student course grades and NBME subject test performance between the preimplementation phase (2020–2021 academic year) and postimplementation phase (2021–2022 academic year) using a 2-sample *t* test, treating the pre-implementation cohort as a historic control. We additionally compared student performance on the neuropathology subcomponent of the NBME subject test. Given the high student performance in the course prior to implementation and the numerous confounding factors that influence student examination performance beyond the neuropathology curriculum, we hypothesized that course outcomes would not change significantly between the preimplementation and postimplementation phases.

### Standard Protocol Approvals, Registrations, and Patient Consents

Administration of the novel survey and conduct of the interviews were reviewed and approved by the local institutional review board; all other course outcomes used in assessing the curricular changes were included as part of typical course conduct.

### Data Availability

Data will be made available through request directed to the corresponding author.

## Results and Assessment Data

Among the class of 104 students, 92 completed the end-of-course evaluation (88.5% completion rate). There was no change in the mean (SD) of lecturer quality between the preimplementation and postimplementation phases as rated on lecturer evaluations, comparing all neuropathology lecturers (4.24 [0.6] vs 4.42 [0.3]; *p* = 0.26) and noncore faculty lecturers (4.05 [0.56] vs 4.26 [0.16]; *p* = 0.22). Student opinions of the TBL sessions were also positive on the end-of-course evaluation, with 79.4% of the 92 students who completed the survey rating the sessions as good or excellent.

**Figure 2:**
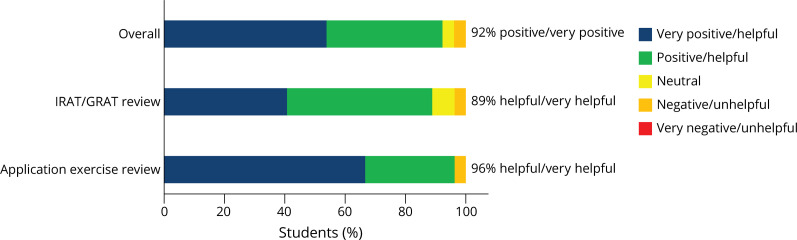
Student Opinions of the Team-Based Learning Sessions GRAT = group readiness assessment test; IRAT = individual readiness assessment test.

Twenty-seven (26.0%) students completed the novel survey on the TBL sessions. On this survey, approximately 90% of students felt the TBL sessions as well as the IRAT/GRAT and application exercise review portions were either helpful or very helpful (Figure 2). Twenty-five (92.6%) respondents felt that the overlap between the lecture and TBL material was appropriate, and 24 (88.9%) and 23 (85.2%) students, respectively, felt that the difficulty of the IRAT/GRAT and application exercises was appropriate. Overall, students felt the presession work and session length were appropriate, though the survey highlighted that students felt the single textbook reading was excessive in length (eTable 1, links.lww.com/NE9/A12).

Of the survey respondents, 14 provided their contact information; we randomly selected 10 respondents to participate in the semistructured interview. Narrative comments on the end-of-course survey and comments in the interviews identified similar themes. In general, students commented most positively on the quality of the TBL syllabi created by the course director and the synergy of learning between the lectures, TBL prework, and TBL sessions. Students specifically commented on how “it felt like there was a single thought process going from the lecture to the pre-work to the TBL sessions.” Additional positive comments included appreciating that a high performance on the IRAT made students eligible for extra credit, rather than making performance part of the course grade. Negative feedback highlighted concerns with differences in length of the prework, again specifically highlighting concerns about the length of the textbook reading. Some students additionally had concerns about the length of the sessions and specifically found the redundancy between the IRAT, GRAT, and quiz review to be excessive. Additional constructive comments included a concern that there was inconsistency in content coverage during the review portions of each session between the 2 large groups. Table 2 includes illustrative quotes of the most prominent positive and negative themes from the feedback and interviews.

**Table 2:** Illustrative Quotes on the Most Prominent Positive and Negative Opinions of the TBL Sessions From the End-of-Course Survey and Semistructured Interviews

Theme	Illustrative quote(s)
Positive comments	
High-quality, concise syllabi	The TBL syllabi created by [course director] were incredibly helpful The prereadings for TBLs that were written by faculty were awesome, and I referred back to these many times
Reinforced learning	TBL was a highlight of planned redundancy with reading followed by lecture I loved the laboratory activities and TBL exercises to supplement the lecture material The TBLs were directly relevant and dealt with material we've learned before
Low-stake quiz performance	I really liked that the TBLs were extra credit points. Otherwise, I believe this would increase stress and competitive atmosphere The stakes of quiz performance felt just right. It was enough to push me to complete the prereading, but not so high to make me worry about it every Tuesday and Thursday
Constructive comments	
Excessive length of textbook reading	The textbook readings were frankly too long, and it was hard to weed through to figure out what was actually important to know I think many people did not read the super long textbook readings that were assigned…
Redundancy of quiz completion and review	Going over all the answers as a large group after we just went through each one as a small TBL group seemed overly redundant Sometimes it just felt like the instructor was reading the answers back to us a third time
Inconsistency of facilitators	I wonder if there may have been discrepancies in opinions in students who learned from [facilitator 1] vs [facilitator 2] I think there were some inconsistencies between information taught… in one room but not the other

Abbreviation: TBL = team-based learning.

Course outcomes were similar between the preimplementation and postimplementation years with slightly higher absolute overall course performance among the 2020–2021 (preimplementation) cohort compared with the 2021–2022 (postimplementation) cohort (Table 3), though the differences were not statistically significant. Similarly, there was no difference when assessing performance on the neuropathology subsection score of the NBME subject test (82.6% vs 83.9%, *p* = 0.36).

**Table 3:** Course Performance Comparison Between the 2020 (Preimplementation) Cohort and the 2021 (Postimplementation) Cohort

	2020 (n = 104)	2021 (n = 104)	*p* Value
Final course grade, mean (SD) %	82.92 (6.24)	82.47 (6.22)	0.61
NBME subject test, mean (SD) %	81.20 (8.22)	80.25 (7.93)	0.37
NBME pathology subsection, mean (SD) %	82.60 (11.2)	83.94 (9.5)	0.36

Abbreviation: NBME = National Board of Medical Examiners.

## Discussion and Lessons Learned

We demonstrate that a conversion to primarily neurologist and neurosurgeon-led lectures and the implementation of a TBL curriculum can be an effective method to deliver neuropathology content in a medical school neuroscience curriculum. Overall, student opinions of the curricular changes were quite positive, specifically highlighting the appreciation of a clinical correlation throughout the neuropathology curriculum, the benefits of applying knowledge in groups, and the synergy between the course sessions and materials. Student performance on internal and standardized examinations was similar in the preimplementation and postimplementation phases, consistent with our hypothesis. However, it should be noted that while the differences were nonsignificant, student performance on the neuropathology subsection of the NBME subject test was higher in the postimplementation cohort despite worse performance on the examination overall among that group; this potentially suggests a positive impact of the novel curricular changes on neuropathology knowledge attainment.

While our study describes the implementation of curricular changes at a single center, we may be able to generalize the feedback and findings here to implementation at other medical schools despite diversity in curricular approaches across neuroscience courses. First, we demonstrate that nonpathologists can effectively deliver neuropathology content, particularly relevant because medical school programs may not have a neuropathologist available to participate in student education, as has been a challenge at our center. Effective neurologist lecturers can be a reasonable alternative or supplement to neuropathologists in a neuropathology curriculum; as described elsewhere in the literature, this may secondarily facilitate early socialization into clinical neurology and reduce neurophobia.^5-7^

Second, we demonstrate the feasibility of implementing a TBL curriculum to deliver neuropathology content and can use this experience to provide guidance for TBL implementation in neuroscience courses more broadly. Student opinions in our course clearly demonstrated the benefit of a curriculum that was developed and overseen by a small team of neurologic educators. The creation of unified lectures, prereading syllabi, and TBL activities was the strength of the new curriculum due to the synergy between materials. As was demonstrated by the striking difference in opinion on the use of textbook prereading, educators should be judicious about the selection and use of external materials in their curricula. However, a potential solution to this could be the curation of a vetted set of materials such as that used in our course, by a national advocacy organization such as the American Academy of Neurology. This could facilitate dissemination of high-quality content across medical school neuroscience courses with minimal work required by local educators to match the content to their curricular needs.

One of the highlighted benefits of TBL more broadly is that sessions can be run with a limited number of educational faculty. Student opinions in our course similarly highlighted this point and suggested that a single effective faculty facilitator may be most appropriate to ensure consistency in content coverage among students. This is particularly relevant for neuroscience course directors in educational settings with limited faculty support for teaching; TBL represents an effective way for a single facilitator to engage a group of 100 students or more.

This work does have some limitations, both in the assessment methods and in its potential for generalizability. First, student response on the novel survey instrument was low with only approximately a 25% response rate. Response bias by those with the most positive or negative opinions of the course activities may have affected the generalizability of the responses to the broader class. In addition, the use of a historical cohort as the comparator with our interventional group does have the potential to introduce bias, particularly surrounding differences in the 2 groups. That said, we felt it was most appropriate to use a “historical control,” given logistical challenges and concerns surrounding equity in randomizing students within a class to 2 separate educational approaches. Finally, the conduct of the survey and semistructured interviews by the course director may have limited student comfort in sharing their true opinions. The anonymous survey completion and conduct of the interviews after all course grades were finalized were intended to mitigate this risk.

The educational setting where these curricular updates were conducted may also limit the generalizability. As described, the neuropathology curriculum operates in one of the most highly rated courses in the medical school curriculum at the University of Rochester, with a large cohort of dedicated neurologic educators available as course faculty. In addition, the course director receives 20% salary support for course coordination and had an additional 5% salary support for the development of this curriculum through a local educational development program. However, if anything, this would suggest that TBL, particularly, may be a more attractive option at centers where education faculty support is low or lecturer quality is more variable, given the ability for a single facilitator to lead the sessions. Furthermore, as noted earlier, the possibility of using vetted and centralized resources could reduce the upfront burden in curricular development and facilitate implementation.

In this study, we demonstrate the feasibility and utility of using clinician lecturers and a TBL curriculum to deliver neuropathology content in a preclinical neuroscience course. Future directions will include implementing improvements in the course locally based on student feedback and consideration of a method to disseminate created course content beyond the University of Rochester. Our work demonstrates that these methods have the potential to enhance neuropathology and neuroscience education more broadly if implemented elsewhere and ultimately to aid in mitigating neurophobia among medical trainees.

## Appendix Authors

**Table:**

Name	Location	Contribution
Christopher G. Tarolli, MD, MSEd	Department of Neurology, and Center for Health + Technology, University of Rochester, NY	Drafting/revision of the article for content, including medical writing for content; major role in the acquisition of data; and study concept or design; analysis or interpretation of data
Ralph F. Józefowicz, MD	Department of Neurology, University of Rochester, NY	Drafting/revision of the article for content, including medical writing for content; major role in the acquisition of data; and study concept or design

## References

[1] Jozefowicz RF. Neurophobia: the fear of neurology among medical students. Arch Neurol. 1994;51(4):328-329. 8155008 10.1001/archneur.1994.00540160018003

[2] Dorsey ER, Constantinescu R, Thompson JP, et al. Projected number of people with Parkinson disease in the most populous nations, 2005 through 2030. Neurology. 2007;68(5):384-386. 17082464 10.1212/01.wnl.0000247740.47667.03

[3] Dall TM, Storm MV, Chakrabarti R, et al. Supply and demand analysis of the current and future US neurology workforce. Neurology. 2013;81(5):470-478. 23596071 10.1212/WNL.0b013e318294b1cf PMC3776531

[4] Association of American Medical Colleges. Association of American Medical Colleges Medical School Graduation Questionnaire: 2016 All Schools Summary Report. AAMC, 2016.

[5] Tarolli CG, Józefowicz RF. Managing neurophobia: how can we meet the current and future needs of our students? Semin Neurol. 2018;38(4):407-412. 30125894 10.1055/s-0038-1666987

[6] Gutmann L, Cahill C, Jordan JT, et al. Characteristics of graduating US allopathic medical students pursuing a career in neurology. Neurology. 2019;92(17):e2051-e2063. 30926683 10.1212/WNL.0000000000007369

[7] Fantaneanu TA, Moreau K, Eady K, et al. Neurophobia inception: a study of trainees' perceptions of neurology education. Can J Neurol Sci. 2014;41(4):421-429. 24878464 10.1017/s0317167100018436

[8] Youssef FF. Neurophobia and its implications: evidence from a Caribbean medical school. BMC Med Educ. 2009;9:39. 19570231 10.1186/1472-6920-9-39 PMC2714502

[9] Koles PG, Stolfi A, Borges NJ, Nelson S, Parmelee DX. The impact of team-based learning on medical students' academic performance. Acad Med. 2010;85(11):1739-1745. 20881827 10.1097/ACM.0b013e3181f52bed

[10] Burgess A, Bleasel J, Haq I, et al. Team-based learning (TBL) in the medical curriculum: better than PBL? BMC Med Educ. 2017;17(1):243. 29221459 10.1186/s12909-017-1068-z PMC5723088

[11] Reimschisel T, Herring AL, Huang J, Minor TJ. A systematic review of the published literature on team-based learning in health professions education. Med Teach. 2017;39(12):1227-1237. 28664760 10.1080/0142159X.2017.1340636

[12] Sibley J, Spiridonoff S. Why TBL Works. Faculty of Applied Science, University of British Columbia, 2014:1-2. Accessed June 12, 2022. byui.edu/documents/instructional_development/larry%20michaelsen/tbl%20why%20and%20how.pdf.

[13] Armstrong P. Bloom's Taxonomy. Vanderbilt University, 2010. Accessed May 31, 2022. https://cft.vanderbilt.edu/guides-sub-pages/blooms-taxonomy/.

[14] Burgess A, van Diggele C, Roberts C, Mellis C. Team-based learning: design, facilitation and participation. BMC Med Educ. 2020;20(suppl 2):461. 33272267 10.1186/s12909-020-02287-y PMC7712595

[15] Pollack AE. The neuroscience classroom remodeled with team-based learning. J Undergrad Neurosci Educ. 2018;17(1):A34-A39. 30618497 PMC6312146

[16] Ng M, Newpher TM. Comparing active learning to team-based learning in undergraduate neuroscience. J Undergrad Neurosci Educ. 2020;18(2):A102-A111. 32848518 PMC7438168

[17] Anwar K, Kashir J, Sajid MR, et al. Implementation of structured team-based review enhances knowledge consolidation and academic performance of undergraduate medical students studying neuroscience. Adv Physiol Educ. 2020;44(2):232-238. 32412386 10.1152/advan.00162.2019

[18] Brich J. Feasibility, acceptance and impact of team-based learning in neurology: a pilot study. GMS Z Med Ausbild. 2013;30(2):Doc20. 23737917 10.3205/zma000863 PMC3671316

[19] Ng M, Newpher TM. Class size and student performance in a team-based learning course. J Undergrad Neurosci Educ. 2021;20(1):A49-A57. 35540942 PMC9053426

[20] Koles P, Nelson S, Stolfi A, Parmelee D, Destephen D. Active learning in a year 2 pathology curriculum. Med Educ. 2005;39(10):1045-1055. 16178832 10.1111/j.1365-2929.2005.02248.x

[21] Team Based Learning Collaborative. 2022. Accessed May 27, 2022. teambasedlearning.org/.

[22] Parmelee D, Michaelsen LK, Cook S, Hudes PD. Team-based learning: a practical guide: AMEE guide no. 65. Med Teach. 2012;34(5):e275-e287. 22471941 10.3109/0142159X.2012.651179

[23] Gray F, Duyckaerts C, de Girolami U. Escourolle and Poirier's Manual of Basic Neuropathology*.* Oxford University Press, 2018.

[24] Kirkpatrick JD, Kirkpatrick WK. Kirkpatrick's Four Levels of Training Evaluation*.* Association for Talent Development, 2016.

[25] Harris PA, Taylor R, Thielke R, Payne J, Gonzalez N, Conde JG. Research electronic data capture (REDCap): a metadata-driven methodology and workflow process for providing translational research informatics support. J Biomed Inform. 2009;42(2):377-381. 18929686 10.1016/j.jbi.2008.08.010 PMC2700030

